# Supplementary material for: Gut and oral microbial compositional differences in women with breast cancer, women with ductal carcinoma in situ, and healthy women
Source: mSystems. 2024 Oct 29;9(11):e01237-24. doi: 10.1128/msystems.01237-24 (PMC11575313; doi:10.1128/msystems.01237-24)
Supplement: Supplemental Figures — Figures S1 to S6. [file msystems.01237-24-s0001.pdf]

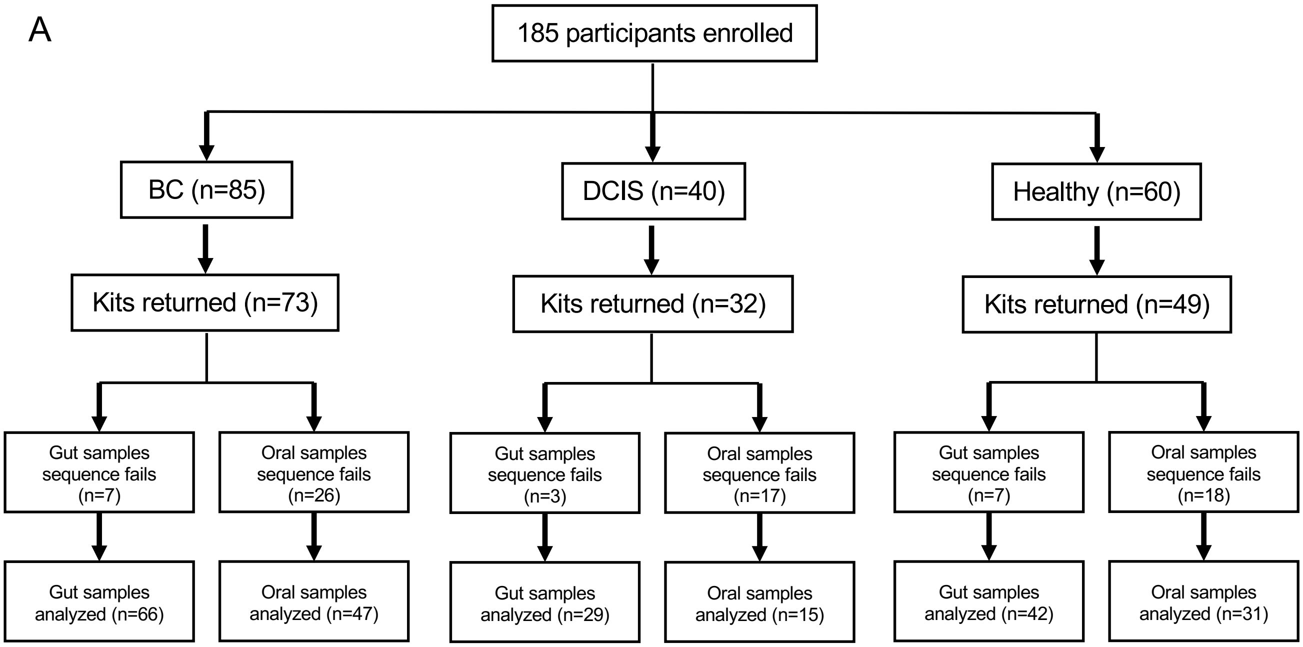


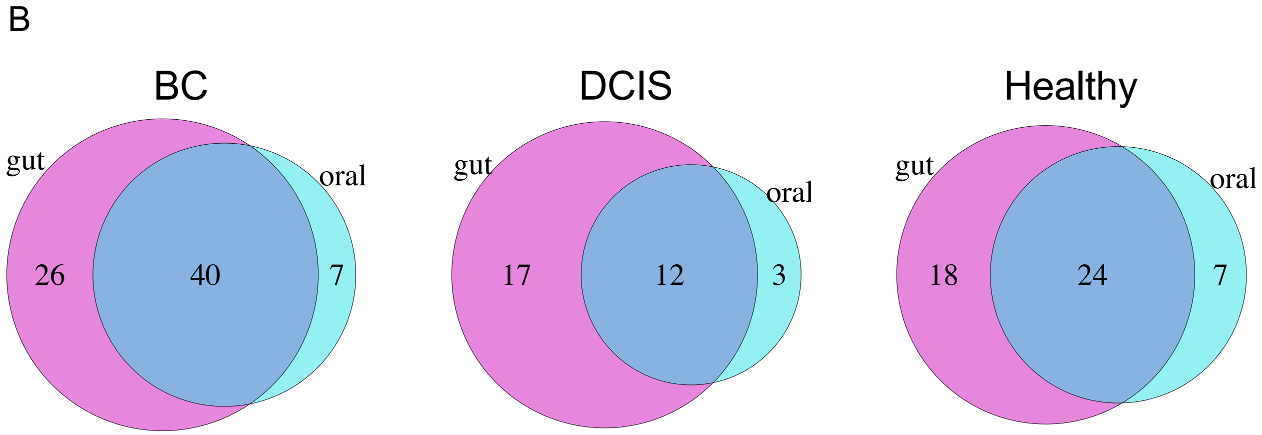


**Figure S1**. (A) Consort diagram showing participant cohorts and flow of samples in the study. (B) Venn diagrams illustrating the numbers of gut (magenta) and oral (light blue) samples analyzed from women with breast cancer (BC), women with DCIS, and healthy women. Overlapping regions indicate number of participants from which there were paired gut and oral samples.

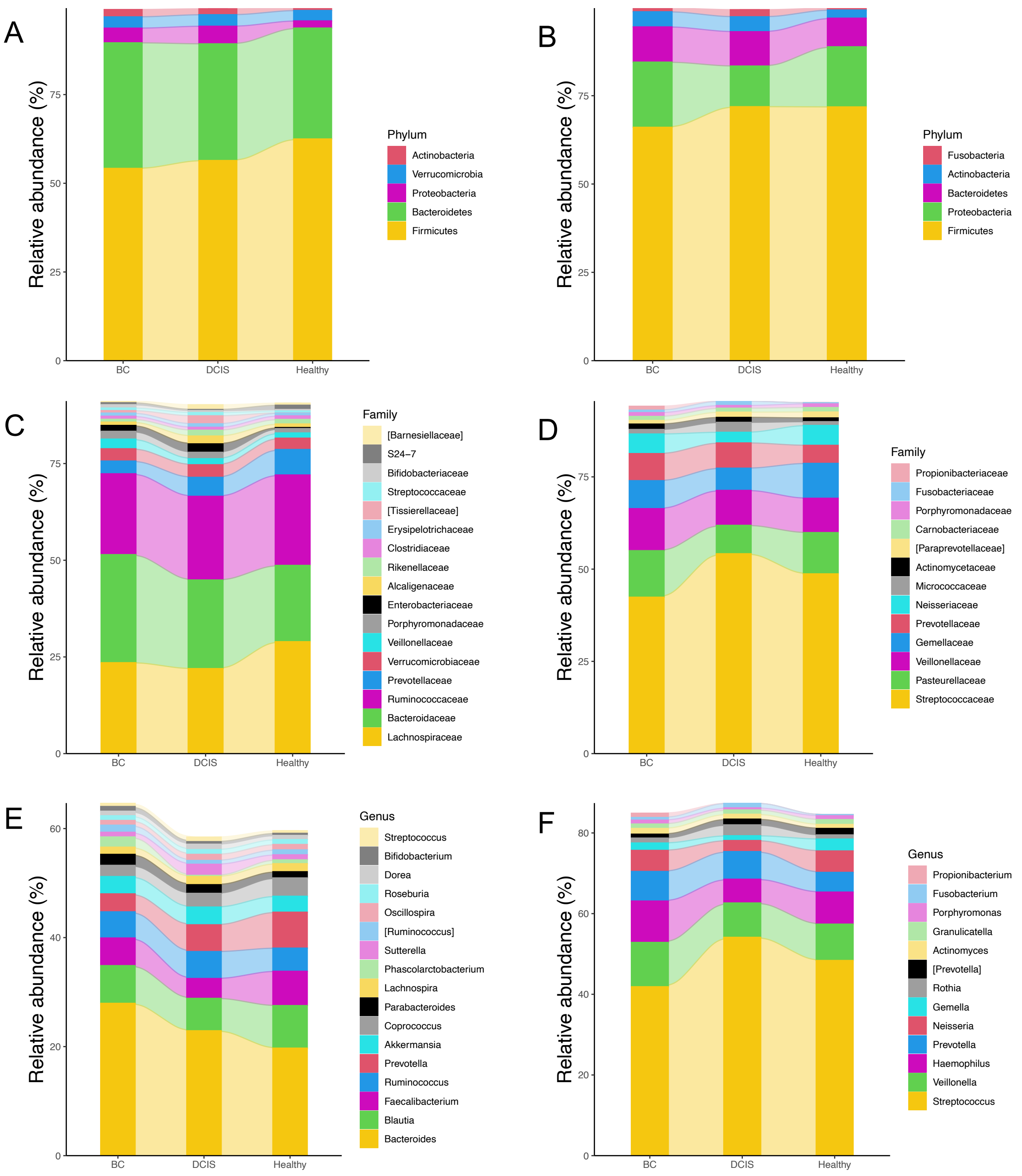


**Figure S2.** Bar charts of relative abundance of bacteria phyla (A, B), families (C, D), and genera (E, F) in gut (A, C, E) and oral (B, D, F) samples. Only taxa with ≥ 1% relative abundance are shown.

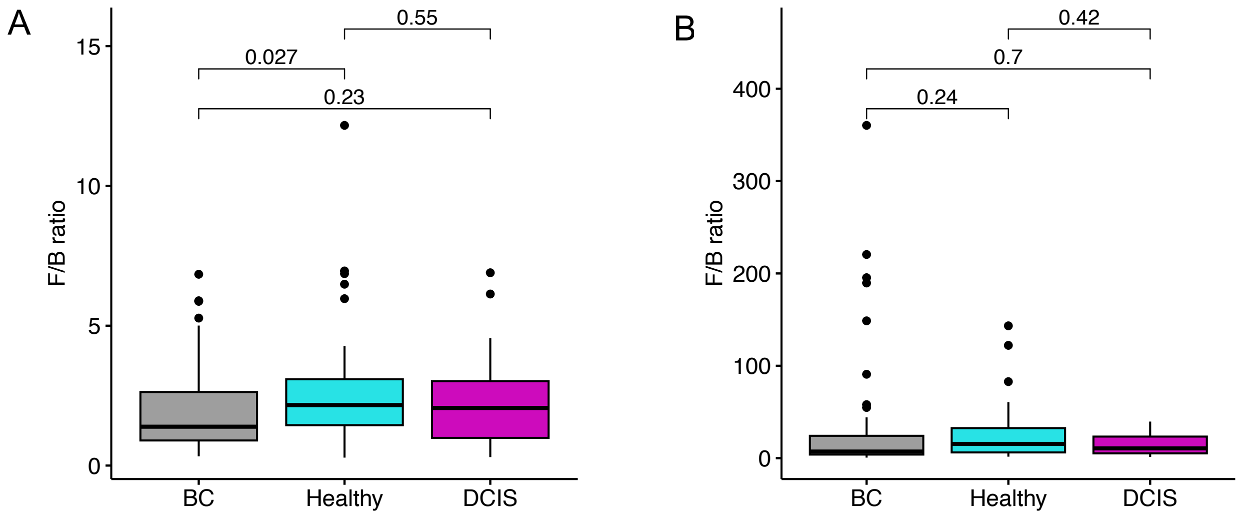


**Figure S3.** Comparison of the Firmicutes/Bacteroidetes ratio between women with BC, women with DCIS, and healthy women. (A) gut microbiota; (B) oral microbiota. p-values calculated using Wilcoxon tests.

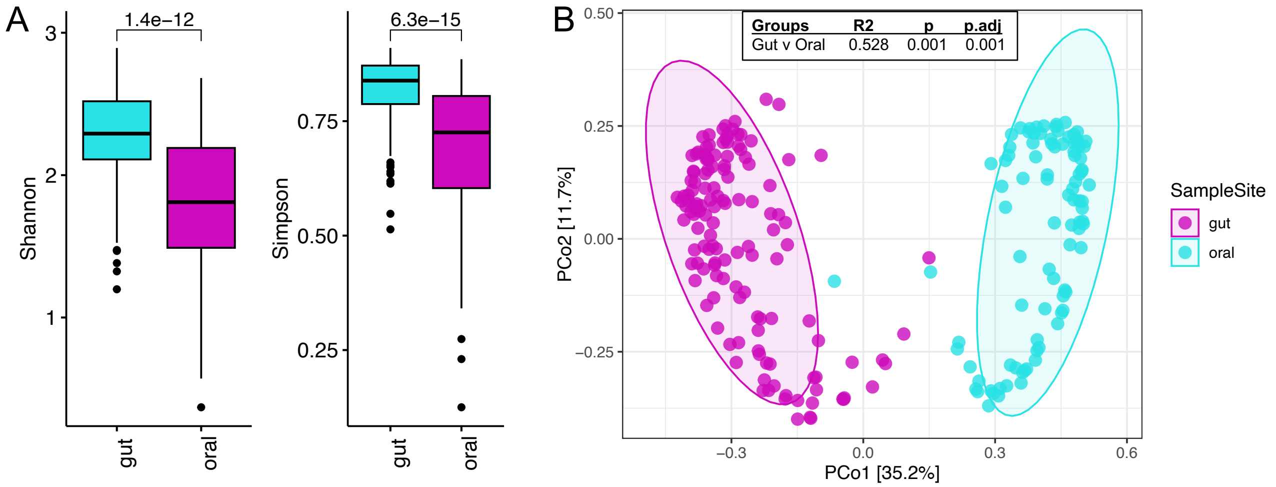


**Figure S4**. Diversity comparisons of gut vs. oral microbiota. (A) Shannon and Simpson alpha-diversity analyses at the genus level. p-values calculated using Wilcoxon tests. (B) Principal-coordinate analysis (PCoA) based on Bray-Curtis distance at the genus level. R2 and p-values calculated using pairwise PERMANOVA.

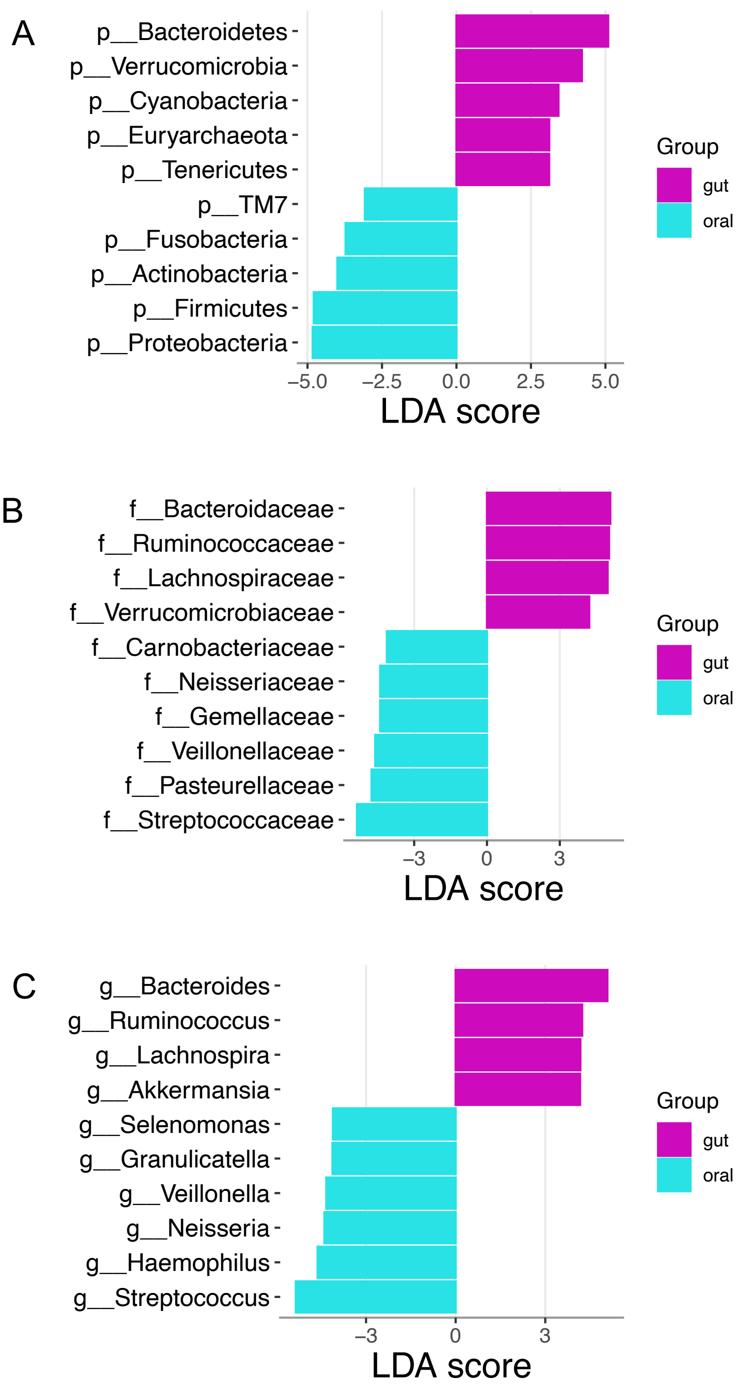


**Figure S5**. LEfSe analysis comparing gut and oral microbiota at the phylum (A), family (B), and genus (C) levels. The top 10 differentially abundant taxa, based on LDA score, are shown.

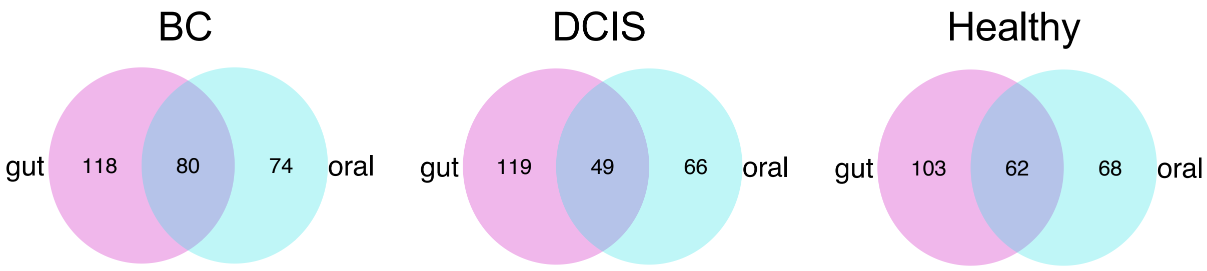


**Figure S6**. Venn diagrams illustrating the number of shared taxa between gut and oral microbiota in each cohort.
